# Survival in patchy landscapes: the interplay between dispersal, habitat loss and fragmentation

**DOI:** 10.1038/srep11898

**Published:** 2015-07-07

**Authors:** Bernardo B. S. Niebuhr, Marina E. Wosniack, Marcos C. Santos, Ernesto P. Raposo, Gandhimohan M. Viswanathan, Marcos G. E. da Luz, Marcio R. Pie

**Affiliations:** 1 Laboratório de Dinâmica Evolutiva e Sistemas Complexos, Departamento de Zoologia, Universidade Federal do Paraná, CP 19020, 81531-980, Curitiba-PR, Brazil; 2 Departamento de Física, Universidade Federal do Paraná, CP 19044, 81531-980, Curitiba-PR, Brazil; 3 Laboratório de Física Teórica e Computacional, Departamento de Física, Universidade Federal de Pernambuco, 50670-901, Recife-PE, Brazil; 4 Department of Physics and National Institute of Science and Technology of Complex Systems, Universidade Federal do Rio Grande do Norte, 59078-970 Natal—RN, Brazil

## Abstract

Habitat loss and fragmentation are important factors determining animal population dynamics and spatial distribution. Such landscape changes can lead to the deleterious impact of a significant drop in the number of species, caused by critically reduced survival rates for organisms. In order to obtain a deeper understanding of the threeway interplay between habitat loss, fragmentation and survival rates, we propose here a spatially explicit multi-scaled movement model of individuals that search for habitat. By considering basic ecological processes, such as predation, starvation (outside the habitat area), and competition, together with dispersal movement as a link among habitat areas, we show that a higher survival rate is achieved in instances with a lower number of patches of larger areas. Our results demonstrate how movement may counterbalance the effects of habitat loss and fragmentation in altered landscapes. In particular, they have important implications for conservation planning and ecosystem management, including the design of specific features of conservation areas in order to enhance landscape connectivity and population viability.

Habitat loss and fragmentation are among the main causes underlying changes in patterns of diversity and distribution of organisms. Such changes have possibly already lead to species extinctions^1^—a problem of great concern. These processes have occurred intensely on both local and regional scales and in almost all biomes around the world^2^. For instance, in the Brazilian Atlantic rainforest, which is considered a biodiversity hotspot, more than 80% of the forest fragments are smaller than 50 ha, with large (1440 m) average distance among patches. Almost 75% of the forest is less than 250 m from the edges^3^. Similarly, in the United States, less than half of the forests are in landscapes with more than 90% cover and approximately 60% are located within 150 m from the edges^4^. Given these numbers, understanding how species interact with the environment and how spatial changes influence species dynamics (and survival) becomes a priority.

A fundamental question in this context is, *what are the best ways of designing conservation areas in order to enhance connectivity and thus population viability?* One of the classic questions in conservation planning are the SLOSS (single large or several small) and FLOSS (few large or several small) problems, also known as the island dilemma: which landscape is more efficient in preserving biodiversity, one with a single (few) large or one with several small habitat fragments? From recommendations of some widely used and empirically supported (but controversial) hypotheses, which mainly take into account species richness, one would expect that the best scenario is an environment with little or no fragmentation and with large or optimal habitat^5^ (see Ref. 6 for a critical review), but exactly why is that and how to quantify the specific situations?

To answer this question, important studies achieved significant advances related to island biogeography^5^,^7^ and metapopulation models^8^,^9^,^10^, although most of these frameworks do not consider explicitly (and simultaneously) spatial features and the locomotion dynamics of the species involved. Studies of landscape and movement ecology^11^ have addressed animal movement processes in patchy landscapes, focusing on the influence of distinct movement strategies on the efficiency of searching for resources^12^,^13^,^14^,^15^ and on population and community consequences of animal movement in changing landscapes^16^,^17^. Nevertheless, few studies have indicated direct implications for conservation (see, however, Refs 18, 19, 20, 21, 22).

Habitat loss and fragmentation strongly influence animal movement patterns, which are intrinsically related to population dynamics. A key idea for studying movement is to transform recorded animal trajectories—e.g., associated with foraging or dispersal—into a sequence of ordered and connected linear displacements (or steps), with the relative orientations of pairs of successive steps yielding an angular shift distribution (i.e. turning angles). Such discretized tracks can then be analyzed via typical techniques of random walk (RW) theory. As a working hypotheses, one can simply assume that there exists well defined spatial scales of motion, so that animals would perform Brownian RWs with normal diffusion^23^. A more complex behavior is one in which the steps are still normally distributed, but with short range correlations for the turning angles^12^,^24^, giving rise to correlated random walks (CRWs). According to the central limit theorem (CLT), the asymptotical dynamics of more complex RWs is still diffusive as long as the correlation lengths and the second moment of the distribution of step lengths remain finite and bounded.

A large number of empirical and theoretical studies have shown that some scale-free properties (i.e., no specific spatial scale in the displacements distribution) are present in the movement of individuals of a large number of species^25^,^26^, resulting in superdiffusion. In this context Lévy walk searches have been reported in many organisms, from small animals like mud snails^27^ (*Hydrobia ulvae*), honeybees^28^ (*Apis mellifera*) and moths^29^ (*Agrotis segetum*), to large animals like albatrosses^30^ (*Diomedea exulans*) and marine fish^31^,^32^.

Lévy flights and walks are characterized by clusters of small steps separated by fewer (but not too rare) long displacements^25^. Mathematically, they correspond to RWs with an uniform distribution of angular turns and step lengths 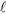 drawn from the family of *α*-stable Lévy distributions (or more generally power law tailed distributions). The Lévy index *α* lies in the interval 0 < *α* ≤ 2. When the second moment diverges, the generalized CLT implies an anomalous (superdiffusive) dynamics. The asymptotically large-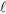 limit of the Lévy distributions is given by a power-law tailed function with exponent *μ* = *α* + 1 > 1. In this contribution we consider a truncated Lévy walk, with step length distribution.
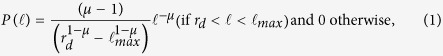
in which *r*_*d*_ denotes a lower cutoff of biological origin (see *Methods*). In contrast with the CRWs, the convergence of the dynamics of a truncated Lévy walk to the Brownian behavior is ultraslow^33^, achieved only after a remarkably large number of steps for large upper cutoff length 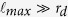 (here we set 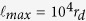; see *Methods*). As a consequence, in the present case the general properties of nontruncated Lévy walks should be retained to a considerable extent and the truncated Lévy walk should sustain a superdiffusive dynamics for a long time before crossing over towards Brownian behavior^34^.

The advantage of Lévy walks is that a single parameter can describe a whole range of movement behaviors^25^, from an area-restricted to a multi-scaled pattern. For *μ* ≥ 3 the RW is statistically similar to a Brownian walk. For *μ* decreasing (within 1 < *μ* < 3) the movement becomes superdiffusive, reaching the ballistic walks limit (straight lines) for *μ* → 1^+^. Theoretical works have pointed out that Lévy walks optimize searches, leading to higher efficiency in finding targets (food or mates, for example) in scarce environments, when compared to some other possible search strategies^25^,^35^. These models have also been used to characterize extinction processes as dynamical phase transitions to absorbing states^36^. The results are not restricted to foraging: they also bear the location of nesting sites, sites non-exposed to predation or new habitat areas in general^12^,^37^,^38^.

The above make clear the importance of combining realistic animal movement models and spatially explicit landscapes structures in order to properly assess the question of individual survival and species decline in fragmented habitats. In a recent contribution^22^, it has been shown that such an approach is already able to quantify how fragmentation *per se* (i.e., minimizing all other ecological effects^39^) can reduce encounter rates. This effect taken isolatedly thus alters other biological interactions and acts as a mechanism that favors population (and therefore also biodiversity) disruption. However, a necessary subsequent analysis requires to determine the specific mechanisms linking the associated decreasing in encounter rates with that of population persistence and species richness. Studies by Wiegand and collaborators^19^,^38^,^40^ have addressed the effects of landscape structure and composition on population dynamics and connectivity using individual-based models. They concentrated on particular species or ecological profiles, with the associated specificities intrinsically taken into account in the results.

In the present contribution we propose a general individual-based model to evaluate which configurations of habitat distribution imply greater individual survival. We consider truncated Lévy walkers searching for habitat in spatially explicit environments, in which we vary independently the levels of fragmentation and total amount of habitat. We go well beyond purely stochastic search and movement-oriented portrayals by including ecological factors in the process. To derive a minimal mathematical formulation, we reduce the mentioned factors to the spatial features of the habitat fragments (e.g., the tendency to stay longer in larger patches of greater carrying capacity) and to the hostile character of the matrix (e.g., higher risks of predation and starvation while traveling from one fragment to another). In this way, our approach addresses a “typical” average situation, representing a global instead of a particular context. Our aim is thus to unveil general mechanisms of action of relevant variables controlling species abundance and population survival in fragmented landscapes (in the sense described, for instance, by Ref. 41). Even considering other ecological processes, our focus is on the influence of between-patch movements on animal survival. Specifically, we wish to understand in which cases different movement strategies can offset the effects of habitat loss and fragmentation.

Through a large number of numerical simulations, we show that not only the total amount of habitat available but also its degree of fragmentation highly influences individual survival rate. Furthermore, less diffusive walkers have lower probability of successfully subsist long travels between patches. We report that the quantitative results strongly rely on the generic ecological factors assumed in the model. Thus, our findings pinpoint and gauge the unfavorable conditions for population abundance caused by fragmented landscapes, as well as illustrate the importance of such type of spatially explicit movement study^42^,^43^,^44^ for an efficient conservation planning^39^,^45^.

## Results

**Figure 1:**
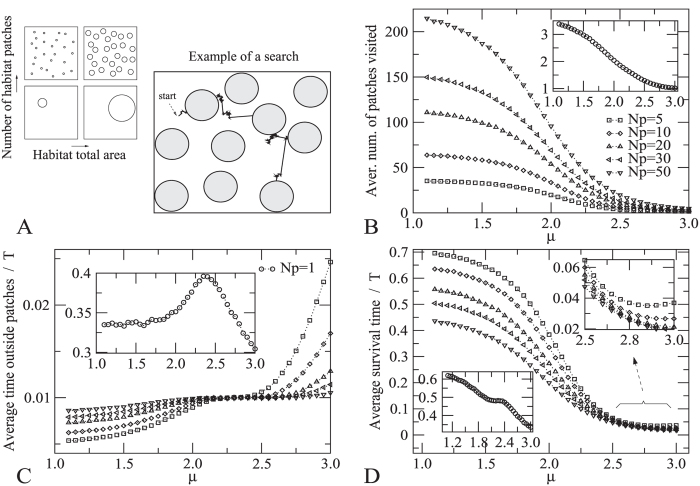
(**A**) Illustration of habitat amounts and fragmentation levels in distinct simulated landscapes. An example of a typical movement dynamics in a search event is shown. (**B**–**D**) Average search quantities versus the movement strategy (represented by the Lévy exponent *μ*) for different fragmentation degrees (from a less fragmented, *N*_*p*_ = 5, to a highly fragmented, *N*_*p*_ = 50), in a scenario in which the amount of habitat is *A*_*H*_/*A*_*T*_ = 10%: (**B**) number of patches visited; (**C**) time outside patches; (**D**) survival time. For comparison, the insets show the case with no fragmentation (*N*_*p*_ = 1).

We performed extensive simulations for distinct parameters values and landscape configurations shown in Fig. 1A. In Fig. 1 we consider the total amount of habitat area (*A*_*H*_) as 10% of the landscape area (*A*_*T*_), but we should also stress that, for habitat areas equal to 1%, 20% and 30% of *A*_*T*_, we have found similar qualitative behaviors. Also, the situation where the number of habitat patches (*N*_*p*_) is 1—shown in the insets of Fig. 1B–D—is useful as a comparison reference since it illustrates the effects of the hostile matrix and movement dynamics when fragmentation is absent (once leaving the sole patch, the searcher can reenter it only after reaching the environment borders—see *Methods*).

The average number of patches visited in a full search event, Fig. 1B, displays an intuitively expected trend: it increases with the number of available fragments *N*_*p*_ and decreases with *μ*, as the diffusivity of the searcher shifts from the ballistic (*μ* → 1) to the Brownian (*μ* → 3) regime, leading to a less efficient habitat exploration. For any *N*_*p*_ > 1, the average total time outside the patches, Fig. 1C, always increases with *μ*, although more slowly in the crossover 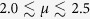 region. For 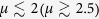 smaller (larger) values of *N*_*p*_ make the total time spent outside the patches to decrease. Indeed, for 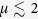 the fragments are more easily found (the searching trajectories are not too ineffectively tortuous) and the time in the matrix region basically depends on the number of travels between patches, which raises with *N*_*p*_. On the other hand, for *μ* approaching 3 the movement tends to normal diffusion and a longer travel time is needed to reach a new patch, specially if the number of fragments is smaller. The interesting behavior in the crossover range 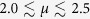 can be understood as a compensation effect: whereas the number of patches visited increases with *N*_*p*_ and decreases with *μ*, the average time to find a new patch decreases with *N*_*p*_ and increases with *μ*; the balance between these trends thus yields a total time outside patches nearly independent of *N*_*p*_ and *μ* in this crossover. Furthermore, at a first sight the decaying behavior in Fig. 1C for *N*_*p*_ = 1 and 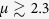 may seem at odds with this previous explanation. However, in this case the low diffusiveness for higher values of *μ* leads the searcher to die frequently along the way, not reentering the patch, and thus decreasing the relative (but not the absolute, observe the ordinate-axis scales for *N*_*p*_ = 1 and *N*_*p*_ ≠ 1 in Fig. 1C) time in the matrix. The average survival time, Fig. 1D, systematically diminishes for greater values of *μ* and *N*_*p*_ (if *N*_*p*_ ≠ 1 it asymptotically saturates for 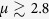, top inset in Fig. 1D). Finally, by comparing Fig. 1C,D we notice that the pattern of the average total time outside the patches does not translate directly into the monotonic decrease with *μ* of the the survival time. In fact, as the probability to die outside patches resets every time a patch is found, the survival time should inversely follow not the total time outside patches, but rather the average time to find a new patch, which actually increases with *μ*, in agreement with the behavior observed in Fig. 1D.

**Figure 2:**
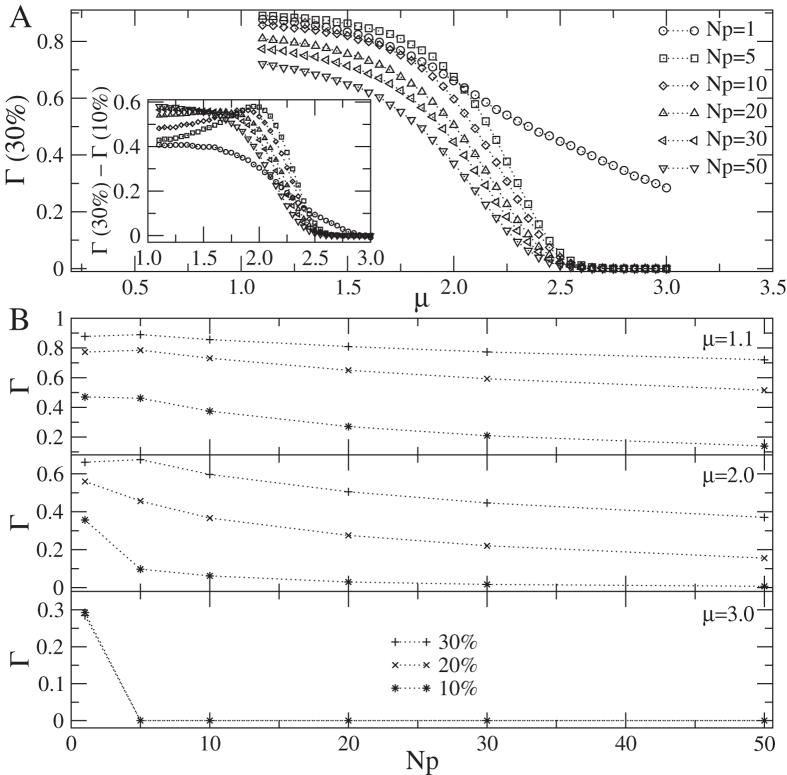
(**A**) Individual survival rate Γ as a function of *μ* for distinct fragmentation levels and total habitat amount *A*_*H*_/*A*_*T*_ = 30%. The inset shows the difference between the cases with *A*_*H*_ = 30% of *A*_*T*_ and *A*_*H*_ = 10% of *A*_*T*_. (**B**) Variation of Γ with the fragmentation level (represented by the number of patches in which a fixed habitat amount is divided, *N*_*p*_) and habitat amount for three distinct diffusive classes of searchers: a nearly-ballistic searcher (*μ* = 1.1), a superdiffusive searcher (*μ* = 2), and a Brownian searcher (*μ* = 3). Note that survival decreases for less diffusive searchers (higher *μ*) and more fragmented landscape (higher *N*_*p*_). For animals with very low dispersal capacity (*μ* ≥ 2.7), survival is possible only when fragmentation is absent (*N*_*p*_ = 1).

Fig. 2A shows a plot of the survival rate Γ versus dispersal strategy parameter *μ* for the total habitat area *A*_*H*_ equal to 30% of the landscape size *A*_*T*_ and distinct numbers *N*_*p*_ of patches. In the inset we show the difference between Γ for habitat area consisting of 30% of *A*_*T*_ and 10% of *A*_*T*_. Notice that the animal survival is boosted as the total amount of habitat increases. Furthermore, as seen in Fig. 2A, the increase in *μ* and in the number of patches (with *N*_*p*_ > 1 and *A*_*H*_ kept fixed) decreases Γ. This decrease in Γ caused by habitat loss, fragmentation and degree of diffusiveness is illustrated in Fig. 2B. The negative impact of habitat fragmentation only (i.e., with both the total amount of habitat *A*_*H*_ and diffusivity *μ* fixed) is enhanced for smaller *A*_*H*_’s and larger *μ*’s. For example, the difference in survivals for *A*_*H*_ = 0.1*A*_*T*_ and *A*_*H*_ = 0.3*A*_*T*_ when *N*_*p*_ = 5 and *N*_*p*_ = 50 are, respectively, (a) *μ* = 1.1: 48% and 80%; and (b) *μ* = 2: 85% and 98%. For animals having a low dispersal rate (*μ* ≈ 3), any amount of habitat fragmentation already can cause a strong impact on survival rate. Indeed, for small *A*_*H*_/*A*_*T*_, the fragmentation of the habitat into 5 patches basically makes no individual able to survive. Hence, even a low level of fragmentation yields drastic changes in the survival when *μ* ≥ 2 (a situation which is true for distinct animal species^22^,^25^).

We can summarize the present results as follows. The survival rate Γ generally decreases with *μ*, regardless of *A*_*H*_ and *N*_*p*_, i.e., it decreases as the dispersal strategy gets less diffusive (*μ* → 3). Moreover, with same conditions for the total amount of habitat and diffusiveness, animals in a less fragmented scenario usually remains less time outside the patches (searching for other fragments), thus living longer. Overall, for all animals and levels of fragmentation, the greater the proportion of habitat in the landscape, *A*_*H*_/*A*_*T*_, the higher animal survival rate Γ.

## Discussion

Our results indicate that habitat loss and movement strategies play a combined influence on the survival rate of individuals in the sense of complexity phenomena in biology and ecology^46^. The different habitat scenarios produce distinct effects on the persistence of individuals, directly related to the basic (hence general) ecological factors considered. We have shown that an increase in the total amount of habitat always results in an increase in the efficiency of the habitat network. This upsurge enhances the probability of finding patches and diminishes the time spent by individuals when traveling among them, thus minimizing exposure to the threats in the matrix. This finding is also in agreement with other theoretical (individual-based and metapopulation models^8^,^9^,^19^,^40^) and empirical works (reviewed by Refs 39,47). For instance, Solé *et al.*^37^ demonstrated that Lévy walk searchers are able to access all the three dimensional space up to a cutoff canopy height in a Barro Colorado Island forest plot. Although represented differently, the environment in their study shows a gradient of habitat amount and connectivity, and is analogue to our approach. As in the present case, Solé *et al.*^37^ reported that species with higher cutoff heights (i.e., with a more connected and greater amount of habitat) present higher search efficiency than species which are only able to explore low forest heights with more sparse and fragmented areas. Furthermore, in a more context-based approach Wiegand *et al.*^19^,^40^ showed that population size increases with habitat amount, a result corroborated by our findings.

Animal movement strategies also play a key role in helping to maintain connectivity and increase survival when fragmentation is imposed on an environment. More diffusive individuals (those with lower Lévy exponent *μ*) present higher survival rates, because they always travel smaller distances while searching for new habitat, Fig. 1. Animals that present *μ* → 1 and live in low fragmented landscapes can almost completely counterbalance the effects of habitat loss by successfully dispersing among patches, which does not happen for Brownian strategies in more fragmented environments. This is due to the greater proportion of long steps in superdiffusive walks when *μ* → 1, which increases the rate of finding patches and decreases the mortality risk in the matrix, if compared to Brownian walks with *μ* → 3, characterized by small displacements and great oversampling of same areas. Higher survival rates for superdiffusive individuals is also related to the assumption of linear increase of dying probability with distance traversed outside patches. In this sense, we expect that a nonlinear dependence of the dying probability with the distance should affect the present results only qualitatively. For example, a faster-than-linear dying rate should generally decrease the survival time for all *N*_*p*_ and *μ*, though still preserving the overall pattern of Fig. 1D.

Studying distinct habitat amounts, mortality rates and landscape configurations, Zollner and Lima^12^ already anticipated that, regarding survival, straighter paths perform better than more sinuous ones. Contrary to our findings, however, they showed that the optimal movement strategy changes from a ballistic walk to a not-so-straight movement as the amount of habitat increases. The reason for this discrepancy arises because they did not consider neither variation in patch size among distinct fragmentation scenarios nor other ecological processes that occur inside patches and prevent animals from dying. These are some aspects that explain why we observed straighter paths as the most efficient strategy in all situations. Zollner and Lima^12^ addressed many of the questions raised here, including ballistic and systematic search strategies and also considered more levels of mortality rate, but did not focus directly on the issue of habitat fragmentation. They have also employed CRWs with Brownian dynamics, instead of truncated Lévy searches. Our study complements their results by probing the influence of (super)diffusive effects at long time scales^24^ and also by including ecological processes that animals perform while residing inside habitat fragments, which are directly related to their survival rate. Solé *et al.*^37^ also unveiled the necessity of lower optimal Lévy exponents for species in scarcer environments, related to smaller cutoff forest heights. Wiegand *et al.*^19^ showed that the association of dispersal ability and enough dispersal habitat can neutralize fragmentation effects.

As for the hostile matrix region, a study by Reynolds^14^ addressed the risk of predation during the foraging activity in homogeneous and patchy environments, introducing the possibility of switching from an extensive to an intensive mode of search upon finding a prey (composite Brownian walk). The resulting search efficiency is then compared with that of a Lévy walker. If the modes of search duration can be chosen so as to optimize the full process efficiency, the Lévy searcher was found to outperform the composite Brownian walker only when the risk of predation is considerable. Indeed, if the risk of predation is low the advantage of switching modes in an efficient way during the search becomes relevant. In Ref. 48 the same author investigated the scenario in which the Lévy walker can use information about patch quality to decide how to search for and within patches. Thus, the optimal strategy for locating patches depends upon how long the walker stays within the patches. Specifically, the smaller patches associated with lower quality acted as “signposts” that help guide the searcher to the most profitable patches. This proposed mechanism hence facilitates the search for high-quality patches, increasing the net energy intake of the walker by reducing its average time spent searching for patches. As described in the *Methods* section, we assume that the time within the patch is proportional to its area, so that in the Ref. 48 context, the Lévy walker would spend less time in smaller patches. But in contrast with Ref. 48, here no assumption is made on the use of the previous history of the walker to predict or indicate its next steps: our searcher can analyzes just minimally the environment features.

When it comes to the actual impact of habitat fragmentation on animal survival and dispersal, we observe different regimes depending on which ecological processes are the dominating driving forces. First, suppose that it would be possible to “turn off” the death rate (here corresponding to set *D* = 0, e.g., by doing the mortality rate *α* = 0; see *Methods*), or just to reduce it to an unimportant level. In this case movement corresponds just to dispersal among habitat fragments. As a consequence, a landscape with several small patches turns to be more suitable to animal survival than one with only a single large patch, since habitat areas are more spread in the environment and the mean distance traveled to find new patches is minimized. This result—directly checked by simulations—brings up a possible adequate mechanism for species dispersal in large scales^38^ and which have not been much emphasized by researchers and conservation managers^49^,^50^: the use of nearby small fragments as ways of crossing larger distances in a fragmented environment. On the other hand, ubiquitous ecological factors as predation, competition, reproduction and death by starvation create the necessity of larger and less fractionated habitats. For instance, the residence time *t* directly depends on the patch size, Eq. 2 (see *Methods*). Thus, since larger values of *t* decrease the time and distance traveled *L* in the matrix region, and death rates *D* also grows with *L*, an intrinsic relation between patch size and survival rate emerges. Indeed, through a general model of metapopulation Etienne and Heesterbeek^10^ have actually shown that distinct fragmentation scenarios do affect the population persistence.

Returning now to the SLOSS and FLOSS problems, we recall some of the suggestions by the classic Diamond^5^ text on a desirable system of natural reserves (although he was actually discussing conditions for maximizing species richness, a similar reasoning can be also applied to species survival in the landscape^47^). Applying the equilibrium theory of island biogeography to a landscape composed by “islands” of habitat in a sea of non suitable habitat, Diamond suggested: (i) considering the total habitat area, a large reserve is better than a small reserve; (ii) from the point of view of fragmentation, “given a certain total area available for reserves in a homogeneous habitat, the reserve should be divided into as few disjunctive pieces as possible” (Ref. 5, p. 144); when it comes to the proximity among reserves, they should be (iii) as close as possible; and (iv) grouped equidistantly from each other rather than disposed linearly. Even facing criticisms^6^,^7^,^49^,^51^, the general ideas proposed by Diamond remains in use, and have been defended as a general truth in conservation planning^49^.

If the main aim is to maintain a landscape with suitable and enough habitat for preserving an still existing ecosystem (greater habitat amount), our results corroborate these suggestions. However, the scenario is quite different when dispersal and colonization become the key issue. Based on our simulation results, there are many aspects to be considered for the island dilemma, and Diamond’s four points above actually do not seem to be a definitive final answer.

Take, for instance, suggestion (ii) above. A larger number of patches (even if at the expense of each being smaller in area) is important to enhance the connectivity of networks or mosaics of fragments, Fig. 1B. Indeed, they diminish the distances traveled between habitat fragments and can be used by animals as stepping stones while migrating among larger habitat areas, specially in heavily fragmented landscapes^38^,^50^. On the other hand, large patches are also important as stable sources of immigrants, increasing the probability of recolonization of fragments that faced local extinctions and providing long-term persistence of animal populations^52^, since they are able to survive for longer times inside large patches (Fig. 2).

Regarding dispersal, proposal (iii) above is valid as long as animals have a limited landscape where they are able to walk and migrate among fragments, or when the network of patches where they need to spread is relatively isolated from other suitable habitat areas. These are exactly the cases in which a greater proportion of long steps is not so efficient as the search strategy^12^. However, if we consider very large scales^53^—which are accessible in our framework due to the periodic boundary conditions (see *Methods*)—suggestion (iii) may not be effective, and patches relatively scattered from each other may constitute the proper pattern. Nevertheless, in both situations one must also consider some other ecological and landscape factors, as well as particular details of the habitat and the species involved, instead of only spatial features of the environment.

Our simulation model was designed to assess the effects of habitat loss and fragmentation on a scale 10,000 times larger than the animal’s radius of perception, i.e., the scale at which the animal sees the landscape as fragmented. However, in real situations when the issue is the human-made fragmentation process, which occurs in scales from hundreds of meters to tens of kilometers^3^, our results show a clear implication: those animal species with area-restricted diffusive movement strategies (here, those which move according to a Lévy exponent *μ* ≥ 2.5) are the most affected, from the point of view of surviving when dispersing among patches. This is the case of small-sized animals like amphibians^54^ and small mammals^17^, which may survive only in small disconnected populations as fragmentation increases. Large-sized mammals and more diffusive animals such as bats and birds are likely not to be strongly affected by habitat loss and fragmentation, in relation to dispersal^17^. However, if we consider that large animals generally require larger home ranges to settle down in a habitat patch, then an overfragmentation may also disturb them, causing them to constantly leave fragments and increasing their mortality risk^17^. About plant seeds, pathogens, and other passively dispersed organisms, mainly those dispersed by animals, we argue that they should be affected in the same way as animals, with the additional complication that the dispersers must be present in the same patches that they are in. Otherwise, or if disperser animals have an area-restricted diffusive behavior, the dispersal of these organisms may be disrupted in the landscape (if they do not perish).

Two final technical comments of biological interest are in order. First, one could ask whether the results obtained in the present study would be different if we had used other kinds of RWs, particularly CRWs. Our conclusions would remain qualitatively the same for time scales shorter than the correlation time (or length) of the CRW, in which a transient superdiffusive behavior is set before the crossover to the long-term Brownian dynamics, according to the CLT^24^,^25^. As discussed, this crossover also takes place for truncated Lévy walks. The difference is that truncated Lévy walks can sustain superdiffusion for very much longer times, in comparison with CRWs^33^. Therefore, if the typical time spent traveling between two patches in a CRW is smaller than its correlation time then its short-term superdiffusive behavior would lead to results comparable to those of a truncated Lévy walk with similar transient dynamics^34^,^55^. Second, real ecological landscapes are much more heterogeneous and complex than binary habitat-matrix landscapes. Here we do not consider other spatial characteristics as habitat quality, aggregation, and matrix quality, by assuming that habitat amount and its spatial distribution are the most important features influencing animal movement and survival^47^. In actual landscapes the fragmentation process often occurs in a non-random way^56^, particularly when they are caused by the human activity. As a consequence, specifically designed networks of habitat patches could emerge as the optimal solution for sustaining survival. In spite of this, qualitatively similar results emerge at least in the cases of scale-free clustered or uniform fragmentation^12^,^15^,^27^. For instance, it was shown experimentally that mud snails present signatures of Lévy searches in environments fully covered by resources, in fragmented landscapes with a regular, random, and fractal distribution of resource patches, and even in environments without any food^27^. This expresses the prevalence of this kind of multi-scaled search behavior in different spatial contexts and supports the approach used here. Besides, they found that in fractal landscapes the average time to find a patch is lower, in comparison to random landscapes, which suggests that in fractal landscapes the survival rate is even higher, mainly for superdiffusive searchers.

In conclusion, by considering a Lévy walk model which incorporates aspects of animal movement in spatially explicit environments we have unveiled important interdependence between habitat fragmentation, movement strategies and the survival rate of animal populations, a proxy for population viability. Although metapopulation and island biogeography models^6^,^7^,^8^,^9^,^10^ have already predicted some of the results reported here, as the increased survival in landscapes with greater amount of habitat, or higher colonization rates when average distance among fragments is lower, they fail in considering movement in an explicitly and detailed manner, as already pointed by Zollner and Lima^12^. Here we provided a step forward and showed in which contexts of habitat loss and fragmentation diffusive and superdiffusive strategies are more efficient in providing landscape connectivity and animal survival. The model allows a simple and straightforward way to include and balance dispersal and connectivity information into the design and management of remaining habitat areas. Our findings corroborate more specific and taxon-based approaches previously published^19^,^38^,^40^. Future studies should incorporate further spatial attributes in the habitat and in the matrix characteristics, such as quality and composition, as well as improved mechanisms of interaction among individuals (e.g. the simultaneous movement of predator and prey and the resulting dynamics) and between individuals and the environment^21^, in order to deal with the theoretical challenge of understanding how the survival rates of animal species are influenced by the impact of habitat fragmentation.

## Methods

We consider habitat loss as the reduction of the total amount of habitat in the landscape and differentiate it from habitat fragmentation, the subdivision of a fixed habitat area in a greater number of patches or fragments (habitat fragmentation *per se*^39^). The simulated environments are two-dimensional landscapes formed only by the habitat and an inhospitable matrix. The total habitat area is constituted of circular patches—a common approximate pattern in large territory scales^4^—inside which animals can mate, reproduce, find food and interact with other individuals. These circular patches are the only locations providing the necessary conditions for survival and persistence. As we are concerned about habitat fragmentation effects, we consider mortality negligible inside fragments, in comparison to the matrix: individuals cannot die inside the patches, only outside. We simulate just the movement of animals searching for habitat among patches: we do not address movements inside the fragments. There are two main reasons for this. First, the richness of behaviors and activities animals perform inside suitable areas lead them to do different kinds of movement (e.g., to mate, sleep, eat and rest). But those movements (although eventually disturbed^42^) are in principle present regardless the existence or not of fragmentation. Since our main interest is on the dynamics taking place in the matrix region, we do not address locomotion within a patch. Second, strong empirical evidences of Lévy walks have been found mainly for foragers on environments with low availability of resources^25^,^57^, and that is actually the case of the space between habitat fragments.

We assume exclusively disturbed landscapes, with the total amount of habitat area (*A*_*H*_) ranging up to 30% of the whole environment (*A*_*T*_). This is indeed the range of habitat amount that theoretical studies suggest that the effects of habitat loss and fragmentation are most intense^39^,^58^. The number of habitat patches *N*_*p*_ varies between no fragmentation (*N*_*p*_ = 1) to a high (*N*_*p*_ = 50) fragmentation degree. The environments (shown in Fig. 1A) are square regions with side *M* = 10^4^*r*_*d*_ (total area 
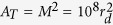
) and periodic boundary conditions (PBC), consisting in torus-like landscapes^59^. All distances are measured relatively to the animal’s radius of detection, *r*_*d*_, a variable that characterizes the range within which animals can detect habitat. One can simulate the way different animals perceive the same level of habitat distribution by varying *r*_*d*_.

Simulated animals perform truncated Lévy walks, since most rigorous statistical approaches have shown that they outperform (nontruncated) Lévy walk models when fitting animal movement data (see, e.g., Refs 27,30), while maintaining superdiffusive properties over large scales^33^. Besides, it has been experimentally verified that some animals may intrinsically perform truncated Lévy searches^27^. Here the truncation (maximum step length) is set to 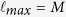, i.e., the landscape size. In each run, the animal’s starting position and the patches centers are randomly chosen from an uniform distribution. For the animal movement, we take the *μ* values from 1.1 to 3.

Our model assesses the influence of habitat spatial configuration on animal survival through three basic aspects: (a) the movement strategy (parameterized by the Lévy exponent), (b) the distinct scenarios of habitat loss and fragmentation, and (c) important ecological processes like predation, competition, and starvation. The model rules are the following. An individual searches for habitat patches. Once a fragment is found, the animal remains in it during a certain residence time *t*. After *t*, the searcher (or one of its descendants) leaves (for different reasons, e.g., competition or resource depletion) the patch and then starts to look for another patch. If, by chance, the searcher returns to the previously left patch before visiting a different one or reaching the environment borders, it does not enter into the fragment. Instead, the searching trajectory just gets specularly reflected from the patch boundary, with the animal thus continuing the search. This dynamics goes on until a maximum simulation time *T*. We also assume a probability *D* for the searcher to die while traveling among patches. By denoting *L* as the distance traveled within the matrix in order to find a new patch, we take *D* = *αL*/*M*, with *α* being a fixed mortality rate. Thus, the more the animal travels in the matrix, the more likely it will perish, e.g., captured by a predator or due to starvation, and this increase in the probability of dying is linear. In our simulations we consider *α* = 0.01 (we have also tested 0.05 with no qualitative changes in the results). Hereafter we call a *full search event* the entire process of an individual performing a whole search run, ended either because the searcher has died along the way (i.e. before *T*) or because it has completed the task at time *T*. If the animal is (is not) able to survive until *T*, a survival (a death) is computed. The mean survival rate Γ, defined as the mean ratio between the number of survivors at the end of the runs and the number of simulations, is thus calculated by averaging over 10^6^ full search events. We take the speed *v* of animals as constant (here *v* = 1). Thus, any travel distance is numerically equal to the corresponding increment of time (where we use *T* = 10^4^). The residence time *t* is proportional to the fragment area *A*_*f*_ according to (*A*_*T*_ = *M*^2^).
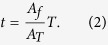


Note that the time spent inside patches is not a constant, but depends on the area of the patch (same as quality in our model), which is what one expects from the marginal value theorem and standard optimal foraging theory^60^. Since we are not interested in the fluctuations in patch size, in any given simulation run all patches have the same area. In such scenario, the marginal value theorem predicts identical time spent inside each patch on average. Given that all the relevant scales of the problem are naturally much larger than *r*_*d*_—otherwise the searching would be trivial (straightforward detection of patches) and the fragmentation would no longer be an issue—the patches are relatively far away from each other. Lastly, for a better characterization of the problem we also compute for a full search event the average number of patches visited along the way, the average time spent outside the patches, and the average survival time.

## Additional Information

**How to cite this article**: Niebuhr, B. B. S. *et al.* Survival in patchy landscapes: the interplay between dispersal, habitat loss and fragmentation. *Sci. Rep.* **5**, 11898; doi: 10.1038/srep11898 (2015).
